# A scaffold-free cartilage construct fabricated using a bio 3D printer accelerates critical-size bone defect regeneration

**DOI:** 10.1016/j.jot.2025.101033

**Published:** 2026-02-28

**Authors:** Hiromu Yoshizato, Daiki Murata, Shohei Kashimoto, Toshihiro Nonaka, Ryota Fujimoto, Yukiko Nagaishi, Manabu Itoh, Tadatsugu Morimoto, Koichi Nakayama

**Affiliations:** a Center for Regenerative Medicine Research, Faculty of Medicine, Saga University, Saga, Japan; b Department of Orthopaedic Surgery, Faculty of Medicine, Saga University, Saga, Japan; c Yoshimura Dental Clinic, Nagasaki, Japan; d Department of Neurology, Takagi Hospital, Fukuoka, Japan; e Department of Thoracic and Cardiovascular Surgery, Faculty of Medicine, Saga University, Saga, Japan

**Keywords:** Adipose-derived stromal cells, Bioprinting, Bone regeneration, Endochondral ossification, Scaffold-free, Tissue engineering

## Abstract

**Background:**

Critical-size bone defects (CSD), often resulting from trauma or tumour resection, represent a challenging clinical condition that is difficult to treat. Although autologous bone grafting is a common treatment, limitations such as donor site morbidity necessitate the development of novel therapeutic strategies. Approaches that mimic endochondral ossification, the natural process of bone development and healing, are increasingly recognised for their bone regenerative potential. The combination of mesenchymal stem cells and scaffolds, used in many bone regeneration studies, has drawbacks, such as scaffold-derived complications including chronic inflammation and fibrosis. To overcome these issues, we used a bio-three-dimensional (3D) printer that enables the fabrication of scaffold-free 3D cellular constructs. This study aimed to establish a novel therapeutic strategy for CSD by generating scaffold-free cartilage constructs from rat adipose tissue-derived mesenchymal stromal cells (rAT-MSCs) and evaluating their regenerative potential.

**Materials and methods:**

Scaffold-free cellular constructs were fabricated using rAT-MSCs. Cartilage constructs were generated by chondrogenic induction. A 5-mm CSD was created in the diaphysis of the rat femur. Three experimental groups were established: a Defect group (n = 9), in which no material was implanted into the defect; an MSC group (n = 9), in which undifferentiated constructs were implanted into the defect; and an MSC-Ch (AT-MSC-derived chondrocyte) group (n = 9), in which cartilage constructs were implanted into the defect. Computed tomography (CT) and histological analyses were performed at 6 and 12 weeks post-implantation.

**Results:**

CT scans showed significantly higher bone volume/total volume ratios in the MSC-Ch group than in the Defect and MSC groups at 6 and 12 weeks (*p* < 0.01). Histologically, the MSC-Ch group exhibited robust formation of new cortical and cancellous bone, continuous with native bone margins, leading to bone bridging. In contrast, the Defect and MSC groups demonstrated new bone formation confined to the periphery of the defect, with the central regions predominantly occupied by adipose and fibrous tissues. Histological scoring supported these findings, with the MSC-Ch group achieving significantly higher scores than the Defect and MSC groups at both time points (*p* < 0.05).

**Conclusion:**

Implantation of scaffold-free cartilage constructs derived from rAT-MSCs effectively promoted CSD healing. To the best of our knowledge, this is the first study to successfully regenerate a long bone CSD using AT-MSCs as the cell source by mimicking the endochondral ossification pathway. However, further studies using larger animal models are required to validate and translate these findings.

**The translational potential of this article:**

Fabricating scaffold-free cartilage from AT-MSCs with a bio-3D printer is a novel solution for bone regeneration, circumventing biomaterial-related complications. This reconstructive method shows promise for various bone defects, including trauma, revision arthroplasty, spinal pathologies, and periodontal disease.

## Graphical abstract

**Figure:**
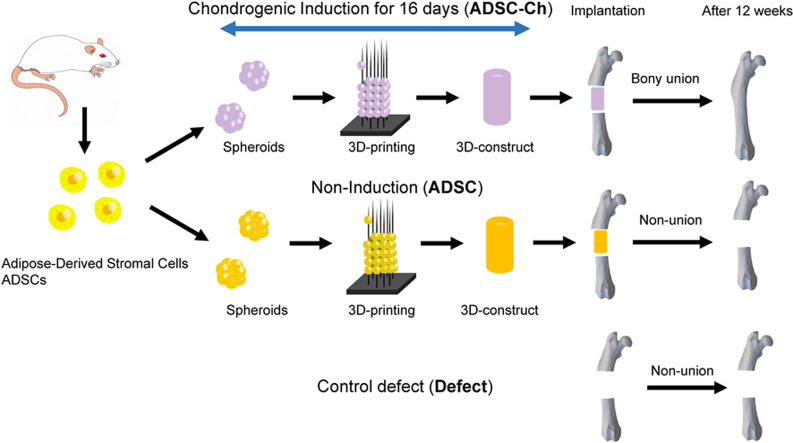

## 1 Introduction

Critical-size bone defect (CSD) is a refractory condition that cannot heal naturally with conventional fracture treatments such as screw or plate fixation. In CSD, significant bone loss occurs due to trauma or tumours [1]. While bone loss occurs in 0.4 % of all fractures, the incidence is particularly high in tibial fractures [1]. Extensive resection associated with bone tumours can also cause bone loss. Although limb amputation can be avoided, 80 % of bone malignancies result in extensive bone loss [2]. CSD does not heal with the fracture treatments and requires therapeutic interventions to promote the formation of blood vessels and tissues for repair [1]. Clinical treatments for CSD include the Masquelet technique, vascularized autogenous bone grafting, free autogenous bone grafting, and bone transport using Ilizarov external fixation. However, each treatment method has potential drawbacks and associated complications [3]. In particular, the Masquelet technique, vascularized autogenous bone grafting, and free autogenous bone grafting require the harvesting of autologous bone. The use of autologous bone is restricted by a limited harvestable amount and is associated with donor site complications, including nerve damage (17.8 %), bacterial infection (1.0 %), and fractures (1.2 %) [4]. To address these issues, bone tissue engineering (BTE) research has been conducted. In studies utilising mesenchymal stem cells (MSCs), methods such as direct injection of MSC suspensions or administration of MSC spheroids into the CSD have been explored [5]. However, when MSC suspensions were introduced into defect sites *in vivo*, over 80 % of the transplanted cells were lost within the first week post-injection [6]. To ensure a more reliable engraftment of cells at the implantation site, implantation methods combining MSCs with scaffolds have been employed. This is because transplanting cells within a scaffold results in higher cell viability compared to injecting them as a cell suspension [7].

As an additional approach to enhance bone formation, recent research has focused on endochondral ossification, which mimics bone development and fracture healing to achieve enhanced bone regeneration [8]. This method has been reported to exhibit superior osteogenic potential; however, a critical requirement is the use of a cell source with high chondrogenic capability. Therefore, most studies mimicking endochondral ossification utilise bone marrow-derived mesenchymal stromal cells (BM-MSCs) or synovial mesenchymal stromal cells (SM-MSCs) as the cell source, owing to their higher inherent chondrogenic potential compared to that of adipose tissue-derived mesenchymal stromal cells (AT-MSCs) [9]. However, numerous studies have shown that the chondrogenic differentiation capabilities of AT-MSCs can be effectively improved through various strategies. These include the addition of growth factors (e.g., transforming growth factor-β3 [TGF-β] and bone morphogenetic protein [BMP]), the application of centrifugal force, and exposure to hypoxic conditions [[10], [11], [12]]. AT-MSCs offer distinct advantages as they can be abundantly harvested from adipose tissue throughout the body and are readily isolated and expanded *in vitro*.

Many studies aiming to mimic endochondral ossification have employed a strategy in which MSCs are induced to undergo chondrogenic differentiation in combination with a scaffold and then implanted into animal models. However, concerns have been raised regarding issues such as chronic inflammation and fibrosis associated with the use of scaffold materials, as well as biofilm formation in cases of bacterial infection [13]. Additionally, controlling the degradation rate of scaffolds is challenging, and scaffold materials may interfere with cell–cell interactions [13]. A bio-three-dimensional (3D) printer capable of creating 3D cellular constructs with arbitrary shapes without scaffolds has been developed [14]. Additionally, bio-3D printing has been successfully used to fabricate various tissues [14], including nerve conduits [15] and cartilage [16], by leveraging a broad spectrum of cell types. This study aimed to develop a novel treatment for CSD by implanting scaffold-free cartilage constructs, fabricated using a bio-3D printer, into a rat femoral defect model.

## 2 Materials and methods

First, we isolated and expanded rAT-MSCs from rat interscapular adipose tissue. Next, spheroids of rAT-MSCs were created using nonadhesive plates, and a scaffold-free cellular construct was fabricated using a bio-3D printer. Chondrogenic differentiation was initiated during spheroid formation. After 14 days of culture, the construct was autologously implanted into the femur of the rat to evaluate its efficacy. The following sections describe each process in detail.

### 2.1 Animals

A total of 30 female Wistar rats (5.5 ± 0.89 weeks old; Charles River Laboratories Japan, Inc., Shiga, Japan) with a mean weight of 149 ± 27.6 g were used to isolate and expand rAT-MSCs. A total of 30 rats underwent implantation and were allocated to specific endpoint analyses as follows: 27 rats were assigned for CT evaluation and subsequent histological scoring, and three rats were designated for non-decalcified histological staining. At the time of implantation, the rats were 13.0 ± 4.3 weeks old and weighed 292.7 ± 50.1 g. All animals were housed under a 12-h dark–light cycle (lights on from 08:00 to 20:00) at 23 ± 2 °C and 50 ± 10 % humidity, with ad libitum access to food and water. All procedures were evaluated and approved by the Institutional Animal Care and Use Committee of Saga university (application no. A2022-017-0). All animal experiments in this study were conducted in accordance with the ARRIVE guidelines.

### 2.2 Isolation and expansion of rAT-MSCs

Intrascapular adipose tissue (0.35 ± 0.25 g per animal) was aseptically collected from anaesthetised animals. General anaesthesia was induced with 5 % isoflurane and maintained with 2.0 % isoflurane (Fujifilm Wako Pure Chemical Industries, Co., Ltd., Osaka, Japan) and 2 mg/kg butorphanol was administered for postoperative analgesia. rAT-MSCs were isolated from the adipose tissue using the method described previously [17]. In brief, the tissue was finely minced and enzymatically digested for 3 h in phosphate-buffered saline (PBS) containing 0.1 % collagenase type I (Worthington Biochemical Corporation, Lakewood, NJ, USA) at 37 °C. The resulting digest was filtered through sterile gauze to remove undigested tissue fragments and then centrifuged at 210×*g* for 5 min at room temperature. The supernatant containing debris and collagenase was carefully removed. The stromal vascular fraction was seeded in primary medium (PM) containing Dulbecco's Modified Eagle's medium (DMEM; Life Technologies, Carlsbad, CA, USA), 10 % foetal bovine serum (FBS; HyClone, Cytiva, Tokyo, Japan), and 1 % antibiotic-antimycotic (Life Technologies) in 10 cm tissue culture dishes (Falcon; Corning Inc., Corning, NY, USA). One week after seeding, the adherent cells were washed with PBS and detached using a recombinant enzyme (TrypLE Select; Life Technologies). These cells were then seeded as passage 1 (P1) cells in a 15 cm tissue culture dish (Falcon, Corning). The rAT-MSCs were expanded in the PM, with medium changes performed twice a week, and passaged at 90 % confluence. At P2, the culture medium was replaced with a cryopreservation medium (Nippon Genetics, Tokyo, Japan), and the cells were stored at −80 °C. All cultures were maintained in a humidified 5 % CO_2_ environment at 37 °C.

### 2.3 Flow cytometric analysis of rAT-MSCs

Flow cytometry was conducted to characterise the surface marker phenotype of MSCs. AT-MSCs at passage 2 (P2) were resuspended in Dulbecco's PBS containing 0.1 % bovine serum albumin (Life Technologies). The cells were then incubated for 30 min at room temperature with fluorescence-conjugated antibodies against various Cluster of Differentiation (CD) markers, including CD45, CD73, CD90, and CD105 (BD Pharmingen Inc., San Diego, CA, USA), following the manufacturer's protocol. An isotype-matched nonspecific antibody (BD) was used as a negative control. Cell fluorescence was acquired and assessed using a flow cytometer (BD Bioscience, San Jose, CA, USA).

### 2.4 Trilineage differentiation assay

The rAT-MSCs were induced to differentiate into adipocytes, chondrocytes, and osteoblasts to investigate their differentiation potential. The details of each differentiations assay are described below.

### 2.5 Adipogenic differentiation assay of rAT-MSCs

rAT-MSCs were seeded at 2.0 × 10^5^ cells/well in 6-well plates (Nunc Cell-Culture Treated Multidishes; Thermo Fisher Scientific, MA, USA) and cultured in PM at 37 °C in a 5 % CO_2_ atmosphere until reaching 90 % confluence. Differentiation was induced by replacing the PM with adipogenic induction medium (Lonza, MD, USA) containing 4.5 g/L D-glucose, 100 μM indomethacin, 10 μg/mL insulin, 0.5 mM 3-isobutyl-1-methylxanthine, and 1 μM dexamethasone, and the cells were cultured for a further 14 days. Following formalin fixation, cells were stained with Oil Red O (Muto Pure Chemicals Co., Ltd., Tokyo, Japan) to evaluate lipid droplet formation. Control cells were cultured in PM for 14 days. The medium was replaced twice weekly.

### 2.6 Chondrogenic differentiation assay of rAT-MSCs

rAT-MSCs were suspended in chondrogenic differentiation medium (CDM; Differentiation Basal Medium-Chondrogenic, Lonza), containing 4.5 g/L D-glucose, 350 μM L-proline, 100 nM dexamethasone, 10 ng/mL TGF-β3 (Peprotech Inc., NJ, USA), and 100 ng/mL BMP-2 (R&D Systems, MN, USA). Spheroids were formed using 96-well non-adherent plates (PrimeSurface 96U Plate; Sumitomo Bakelite, Tokyo, Japan). The rAT-MSCs suspension in CDM was dispensed into each well at a concentration of 1.2 × 10^4^ cells/100 μL. Cells were cultured for 14 days at 37 °C with 5 % CO_2_, and the medium was changed twice a week. After 14 days, cells were fixed with formalin. Frozen sections with a thickness of 8 μm were prepared using a cryostat (Leica CM1950; Leica Biosystems, Nussloch, Germany). Sections were then stained with Safranin O (Muto Pure Chemicals). As a control, rAT-MSCs were suspended in a 1:1 mixture of endothelial cell growth medium (EGM-2 BulletKit; Lonza) and fibroblast growth medium (FGM-2 BulletKit, Lonza), and added to each well at a concentration of 4.8 × 10^4^ cells/100 μL.

### 2.7 Osteogenic differentiation assay of rAT-MSCs

For osteogenic differentiation, rAT-MSCs were seeded in a 6-well culture plate (Nunc, Thermo Fisher Scientific) at an initial density of 2.0 × 10^4^ cells per well using PM as the culture medium. Upon reaching 90 % confluence, the medium was replaced with osteogenic induction medium (Differentiation Basal Medium-Osteogenic, Lonza) containing 100 μM ascorbic acid, 10 mM β-glycerophosphate, and 1 μM dexamethasone. The cells were cultured for 28 days, and the medium was changed twice per week. After 28 days, the cells were fixed with formalin. The samples were then stained with Alizarin Red (Fujifilm Wako) to evaluate calcium deposition. For the control group, rAT-MSCs were cultured in 6-well plates (Nunc, Thermo Fisher Scientific) with PM for 28 days.

### 2.8 Fabrication of a scaffold-free 3D cartilage construct

Scaffold-free 3D cartilage constructs were fabricated as previously reported [18]. The details of the fabrication process were provided below.

### 2.9 Forming chondrogenic spheroids

Cryopreserved rAT-MSCs were expanded in 15 cm dishes (Falcon, Corning) using mesenchymal stem cell growth medium (MSCGM; Lonza) containing 5 ng/mL basic fibroblast growth factor (bFGF; FUJIFILM Wako) and passaged until P4. rAT-MSCs were seeded at a density of 1.2 × 10^4^ cells/well in 96-well non-adherent plates (Sumitomo Bakelite). The medium was then replaced with CDM containing 10 ng/mL TGF-β3 and 100 ng/mL BMP-2. The spheroids were incubated at 37 °C in a 5 % CO_2_ atmosphere. The spheroid diameter was measured using the camera of a bio-3D printer (Regenova; Cyfuse Biomedical Co., Ltd., Tokyo, Japan) on days 1–12 and 15.

### 2.10 Fabrication of a scaffold-free 3D cartilage construct

**Fig. 1:**
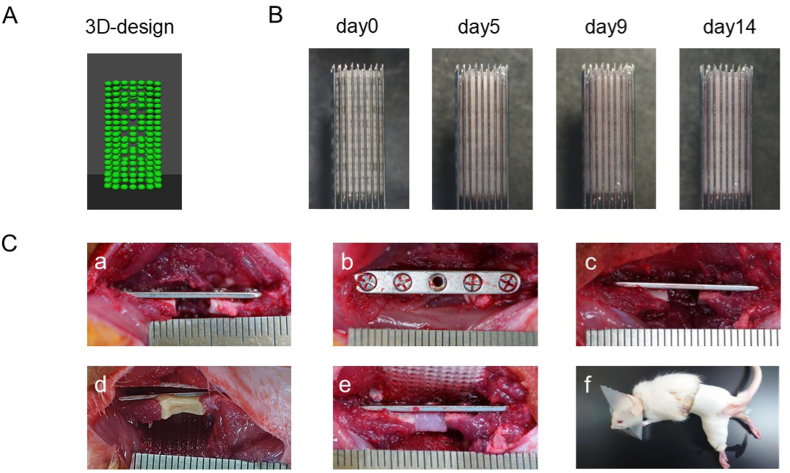
3D-design, fabrication, and implantation of scaffold-free cartilage constructs. (A) A schematic of the computer 3D-design illustrating the arrangement of spheroids. (B) Macroscopic observations of construct formation from spheroid fusion on the microneedles at day 0, 5, 9 and 14. (C) Creation of a 5 mm long circumferential bone defect model and implantation of various constructs. (C-a) Side view of a 5 mm circumferential bone defect created in the femoral diaphysis and stabilised with a plate. (C-b) Top view of the bone-defect model. (C–c) No construct was implanted into the bone defect (Defect group). (C-d) An undifferentiated construct was implanted into the bone defect (MSC group). (C-e) A cartilage construct was implanted into the bone defect (MSC-Ch group). (C-f) The implanted site was protected with a cast bandage for 2 weeks post-implantation. An Elizabethan collar was placed on the rat to prevent it from picking at the cast. MSC, Mesenchymal stromal cell; MSC-Ch, Mesenchymal stromal cell-derived chondrocyte.

The fabricated spheroids reached a diameter of 500–600 μm after 2 days of culture, confirming their suitability for bio-3D printing. The spheroids were then placed onto a fine needle array (hollow 9 × 9 Kenzan; Cyfuse Biomedical) using a bio-3D printer and printed based on a pre-existing design using 3D designer software (Cyfuse Biomedical), as described in a previous study [14]. In this process, the spheroids were automatically picked up from a 96-well plate and placed onto the needle array according to the 3D design. The resulting construct was a cylindrical cellular structure (3.5 mm diameter, 1.8 mm inner lumen, 5 mm length), with a printing time of approximately 1 h per construct. Approximately 350 spheroids were used to fabricate each construct. The 3D computational design specifying the arrangement of spheroids employed for construct fabrication is presented in Fig. 1A. This design incorporates multiple channels to ensure thorough medium perfusion [19], which promotes uniform chondrogenic differentiation throughout the construct. After stacking, the constructs were matured in a bioreactor connected to a peristaltic pump (MasterFlex L/S; Masterflex, IL, USA) to circulate the medium within the constructs. The bioreactor system utilised silicone tubing (3.1 mm inner diameter, 6.5 mm outer diameter) with a flow rate of 200 mL/h. The needle array was removed from the constructs after 14 days and the medium was replaced twice weekly. Fig. 1B shows the fusion of spheroids to form a single construct after stacking. The microchannels gradually became occluded.

Undifferentiated constructs were used as controls. rAT-MSCs were suspended in a 1:1 mixture of Endothelial Cell Growth Medium-2 BulletKit and Fibroblast Growth Medium-2 BulletKit (Lonza) and seeded into each well at a concentration of 4.0 × 10^4^ cells/100 μL. Two days after spheroid formation, the constructs were fabricated using a bio-3D printer in the same manner. The constructs were cultured in a 1:1 Endothelial Cell Growth Medium-2 BulletKit/Fibroblast Growth Medium-2 BulletKit (Lonza) mixture [14]. All the other culture conditions were identical to those used for the cartilage constructs described above.

### 2.11 Scanning electron microscopy

The surface morphology of the 3D-printed cartilage constructs was observed using scanning electron microscopy (SEM). Before observation, samples were trimmed a suitable size, freeze-dried, and then sputter-coated with platinum-palladium. The samples were examined using an S-3400N (Hitachi High-Technologies Corp., Tokyo, Japan) at an accelerating voltage of 5 kV.

### 2.12 Evaluation of mechanical properties of cartilage constructs

The compressive strength of the cartilage constructs was measured using a digital force gauge (ZTA-50 N, IMADA, Aichi, JAPAN). For the measurement, the tubular constructs were incised longitudinally and flattened. A flat compression attachment with a 2.0 mm diameter was then used to compress the construct wall until it ruptured. The compressive strength was defined as the peak force at the point of failure. The Young's modulus was calculated from the slope of the initial linear region of the stress–strain curve.

### 2.13 CSD model creation and construct implantation

**Fig. 2:**
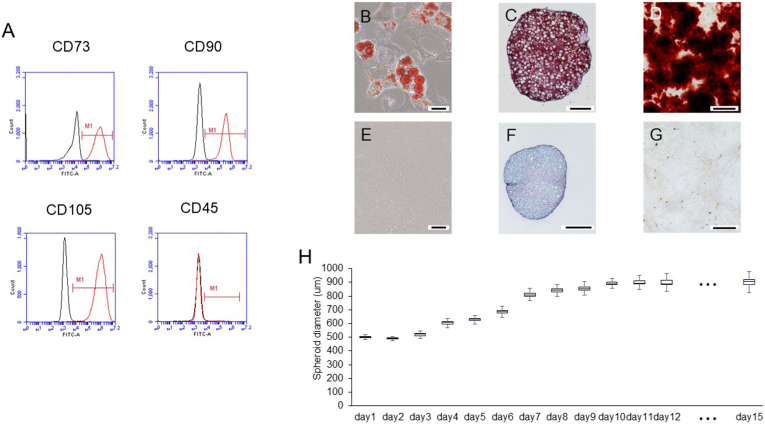
Flow cytometry, tri-lineage differentiation of rAT-MSCs and rAT-MSC spheroid size change after chondrogenic induction (A) Flow cytometry analysis of the isolated rAT-MSCs. (B) Adipocyte-like cells containing small lipid vesicles stained positive for Oil Red O after adipogenic induction. (C)Chondrogenically induced spheroid stained red with Safranin O Control stained with Oil Red O. (D) Calcium apatite stained with Alizarin Red after osteogenic induction. (E) Control stained with Oil Red O. (F) Control spheroid stained with Safranin O and Fast Green. (G) Control stained with Alizarin Red. (H) Time course of spheroid size change during chondrogenic differentiation. Scale bars = 20 μm (B, E) and 200 μm (C, D, F, G). CD, Cluster of Differentiation; rAT-MSC, rat adipose tissue derived stromal cell.

A 5 mm long femoral defect was created in the left femur of the rats, from which rAT-MSCs were harvested for construct fabrication. The constructs were then implanted into the same rats to represent autologous implantation. General anaesthesia was induced with 5 % isoflurane and maintained with 2.0 % isoflurane (Fujifilm Wako); 2 mg/kg butorphanol was administered for postoperative analgesia. Surgery was performed after aseptic draping using clean, sterilised sheets. A 4 cm longitudinal incision was made on the lateral aspect of the left hind limb and the fascia was incised to expose the femur. A 5 mm long CSD was created in the mid-diaphysis of the femur using a sagittal bone saw. The defect was stabilised with a metal plate and four 1.3 mm diameter screws (Toy Cuttable Plate System; Mizuho Medical Co., Ltd., Tokyo, Japan) bridging the defect (Fig. 1C–a and C-b). The rats were assigned to three groups: 1) a defect control group with no construct implantation (Defect group; Fig. 1C–c); 2) a group receiving an uninduced rAT-MSCs construct (MSC group; Fig. 1C and d); and 3) a group receiving a construct composed of rAT-MSC derived chondrocytes (MSC-Ch group; Fig. 1C–e). Regardless of whether a construct was implanted, a nonabsorbable synthetic mesh (PROLENE mesh; Ethicon, Johnson & Johnson, NJ, USA) was wrapped around the bone defect site and fixation plate. This mesh prevented the displacement of the construct and inhibited the intrusion of the surrounding muscle tissue into the defect site. The fascia and skin were closed using nylon sutures. To protect the implantation site, external fixation was performed using a Scotchcast Plus Casting Tape (3M, MN, USA) for a two-week period. Furthermore, an Elizabethan collar was fitted to each animal to prevent ingestion of the cast material or other damage to the cast (Fig. 1C–f). At scheduled time points for computed tomography (CT) and histological evaluation, the rats were euthanised via high-concentration carbon dioxide inhalation. The femurs were harvested for analysis.

### 2.14 Micro-CT

The dissected rat femur was scanned using micro-CT (3D Micro X-ray CT Lab GX130; Rigaku Co., Ltd., Yamanashi, Japan, and SkyScan 1276 micro-CT; Bruker, MA, USA) with the following parameters: tube voltage, 90 kV; tube current, 61 μA; field of view, 60 mm; and high resolution (9 μm/pixel). The 3D images were created using the accompanying image reconstruction software. Quantitative analysis of the bone defect was performed using VGSTUDIO MAX version 3.5.0 (Volume Graphics, Aichi, Japan) to measure the bone volume/total volume (BV/TV) and bone mineral density (BMD).

### 2.15 Histological analysis

Cartilage constructs were fixed in neutral-buffered 10 % formalin for 2 days for histological analysis. The samples were embedded in paraffin, and 5 μm sections were prepared and stained with haematoxylin-eosin (H&E), Safranin O and Fast Green (SOFG), and Masson's trichrome (MTC). These sections were also used for the immunohistochemical staining of type I and type II collagen. Goat polyclonal anti-type I collagen antibody (1310-01; Southern Biotechnology Associates Inc., AL, USA) was used as the primary antibody for type I collagen, and goat polyclonal anti-type II collagen antibody (1320-01; Southern Biotechnology Associates Inc.) was used as the primary antibody for type II collagen. Apoptotic cells within the constructs were detected by a TUNEL assay using an apoptosis detection kit (Merck Millipore, Darmstadt, Germany).

Similarly, rat femurs were fixed in 10 % neutral buffered formalin and embedded in paraffin. 5 μm sections were then prepared and stained with H&E, SOFG, and MTC. The histological sections were evaluated using the quantitative scoring method described previously [20]. Briefly, each specimen underwent histomorphometric analysis, and scores were assigned to four categories based on the percentage of the original bone defect (OBD): newly formed bone (NB), cartilage (CA), fibrous tissue (FT), and remnant defect (RD). The scores from each category were then summed to yield a final score ranging from 0 (no bone healing) to 40 (complete bone healing). Quantitative analysis was performed using a light microscope (BZ-X710, Keyence, Neu-Isenburg, Germany) and image analysis software (BZ-H3A, Keyence). The OBD, NB, CA, and FT areas were identified, delineated, and measured in square millimetres (mm^2^) using the ‘Hybrid Cell Count’ function in the image analysis software. The RD area was calculated by subtracting the NB area from the OBD area. Blood vessels were identified as luminal structures containing at least one erythrocyte (Supplementary Fig. 1) and were counted across the entire defect area. Vessel density was calculated by dividing the total vessel count by the defect area and is expressed as vessels/mm^2^.

### 2.16 Undecalcified stained sections

Calcein (Dojindo Laboratories, Kumamoto, Japan) was used for fluorescent labelling. The dye was dissolved in 2 % sodium bicarbonate aqueous solution (FUJIFILM Wako) to prepare an 8 mg/mL solution. This calcein solution was subcutaneously injected at a dose of 0.1 mL per 100 g of rat body weight. Forty-eight hours after injection, the rats were euthanised, and the femurs were harvested and fixed in 10 % neutral-buffered formalin. The samples were dehydrated, embedded in methyl methacrylate, sectioned (6 μm), and stained using the Villanueva–Goldner method. The sections were dehydrated, cleared, and mounted. Quantitative analysis was performed using a microscope (BZ-X710; Keyence) and image analysis software (BZ-H3A; Keyence).

### 2.17 Statistical analysis

All numerical data are presented as mean ± standard deviation. CT and histological scoring data were compared among the three groups using one-way analysis of variance with Tukey's post hoc test. Pearson's correlation coefficient was used to assess the correlation between vessel density and the results of CT analysis and histological scoring. All statistical analyses were performed using JMP Pro, version 17.2.0 (SAS Institute Inc., Cary, NC, USA). Statistical significance was set at *p* < 0.05.

## 3 Results

### 3.1 Flow cytometric analysis of rAT-MSCs

Flow cytometry revealed that the rAT-MSCs were positive for the MSC markers CD73, CD90, and CD105, but negative for the hematopoietic stem cell marker CD45 (Fig. 2A). These findings are consistent with previous reports [10].

### 3.2 Trilineage differentiation assay of rAT-MSCs

The results of the trilineage differentiation of rAT-MSCs are presented in Fig. 2B–G. Oil Red O staining of adipogenic rAT-MSCs revealed reddish oil droplets throughout the cytoplasm (Fig. 2B). These oil droplets confirmed differentiation of rAT-MSCs into adipocytes. In contrast, uninduced rAT-MSCs showed no oil droplet formation and exhibited negative staining (Fig. 2E). Following chondrogenic induction of rAT-MSCs spheroids, large oval cells surrounded by a Safranin O-stained cartilage matrix were observed (Fig. 2C). Conversely, uninduced spheroids showed negative staining (Fig. 2F). For the osteogenic induction of rAT-MSCs, intense Alizarin Red staining was observed on the basal surface of the well surrounding the cells (Fig. 2D), indicating mineral deposition. In contrast, uninduced rAT-MSCs showed no Alizarin Red staining (Fig. 2G).

### 3.3 Forming chondrogenic spheroids

Spheroids of rAT-MSCs subjected to chondrogenic induction showed a time-dependent increase in size, with the average size increasing from 499.9 ± 8.8 μm on day 1 of culture initiation to 903.6 ± 37.6 μm on day 15 (n = 8, Fig. 2H).

### 3.4 Fabrication of a scaffold-free 3D cartilage construct

**Fig. 3:**
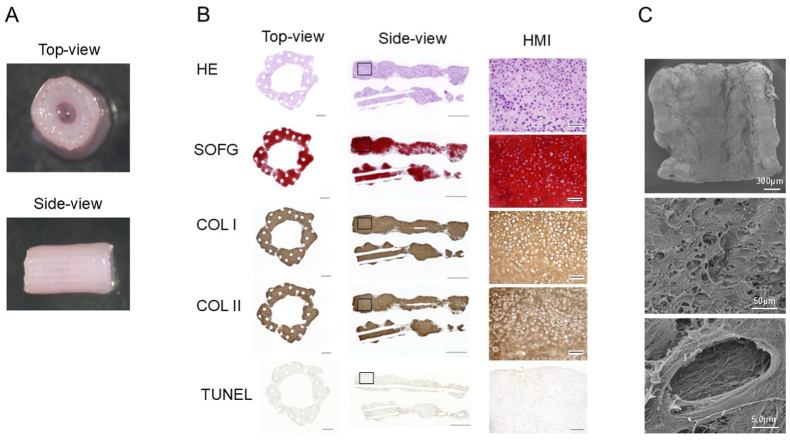
Gross appearance, histological findings, and SEM images of cartilage constructs. (A) The gross appearance of the cartilage construct. (B) Histological findings of the cartilage constructs. Scale bars = 500 μm (top view); 1 mm (side view); 100 μm (HMI). (C) SEM images. COL I, collagen type I immunostaining; COL II, collagen type II immunostaining; H&E, haematoxylin and eosin staining; HMI, high-magnification image; SEM, Scanning Electron Microscopy; SOFG, Safranin O and Fast Green staining.

The engineered cartilage construct was evaluated for its macroscopic appearance (Fig. 3A) and histological features (Fig. 3B). Macroscopically, the construct appeared pale pink and exhibited sufficient elasticity to maintain its shape upon retrieval from Kenzan. Histological analysis of the cultured constructs revealed globally positive staining for SOFG and positive immunostaining for type II collagen. Moreover, at a higher magnification, cells resembling native chondrocytes, characterised by an oval morphology and abundant cytoplasm, were embedded within the extracellular matrix. Although positive immunostaining for type I collagen was also observed, this may be attributed to the short culture period of only 2 weeks. This may reflect the persistence of residual type I collagen secreted by MSCs prior to their full chondrogenic differentiation. Although TUNEL staining revealed a few positive cells, the overall apoptotic rate within the construct was minimal, a finding consistent with previous reports [18].

### 3.5 Scanning electron microscopy

SEM of the 3D-printed cartilage construct revealed a dense, intricate fibrous network on its surface, along with lacunae of varying morphologies (Fig. 3C). These features closely resembled those of native cartilage [21].

### 3.6 Evaluation of mechanical properties of cartilage constructs

The compressive strength of the cartilage constructs was 2.2 ± 0.33 MPa, and the Young's modulus was 1.1 ± 0.16 MPa (n = 4). These results indicate that the constructs possess mechanical properties similar to those of native cartilage [22]. Handling of the construct is shown in the Supplemental Video, demonstrating its elasticity.

### 3.7 Micro-CT

**Fig. 4:**
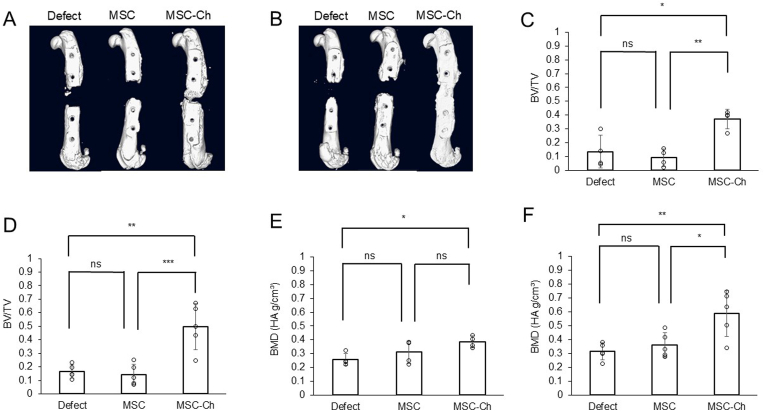
3D-CT images, BV/TV and BMD at 6 and 12 weeks post-implantation (A) 3D-CT image at 6 weeks post-implantation. (B) 3D-CT image at 12 weeks post-implantation. (C) BV/TV at 6 weeks post-implantation. (D) BV/TV at 12 weeks post-implantation. (E) BMD at 6 weeks post-implantation. (F) BMD at 12 weeks post-implantation. ∗*p* < 0.05, ∗∗*p* < 0.01. 3D-CT, three-dimensional computed tomography; BMD, bone mineral density; BV/TV, bone volume/total volume; MSC, Mesenchymal stromal cell; MSC-Ch, mesenchymal stromal cell-derived chondrocyte; ns, non-significant.

Fig. 4A and B shows representative 3D-CT images at 6 and 12 weeks post-implantation, respectively. At 6 weeks post-implantation, both the Defect and MSC groups exhibited new bone formation that was limited to the original bone resection areas. In contrast, the MSC-Ch group demonstrated partial osseous bridging formed by new bone. At 12 weeks post-implantation, new bone formation in the Defect and MSC groups was observed only in limited areas. In contrast, in the MSC-Ch group, most bone defects were replaced with new bone. The 3D-CT results at 24 weeks post-implantation are shown in Supplementary Fig. 2A. The BV/TV results for each group at 6 and 12 weeks post-implantation are shown in Fig. 4C and D, respectively. At 6 weeks post-implantation, the BV/TV was 0.13 ± 0.12 in the Defect group, 0.09 ± 0.06 in the MSC group, and 0.37 ± 0.07 in the MSC-Ch group (n = 4 per group; *p* = 0.004). By 12 weeks post-implantation, the respective BV/TV values were 0.17 ± 0.05 in the Defect group, 0.14 ± 0.17 in the MSC group, and 0.50 ± 0.17 in the MSC-Ch group (n = 5 per group; *p* < 0.001). As shown in Fig. 4E and F, the bone mineral density (BMD) at 6 weeks post-implantation was 0.26 ± 0.04 g/cm^3^ in the Defect group, 0.31 ± 0.08 g/cm^3^ in the MSC group, and 0.39 ± 0.04 g/cm^3^ in the MSC-Ch group (n = 4 per group; *p* = 0.004). At 12 weeks post-implantation, the BMD was 0.31 ± 0.06 g/cm^3^ in the Defect group, 0.36 ± 0.09 g/cm^3^ in the MSC group, and 0.59 ± 0.17 g/cm^3^ in the MSC-Ch group (n = 5 per group; *p* = 0.005). These findings indicated that the MSC-Ch group achieved the most substantial new bone formation at both time points.

**Fig. 5:**
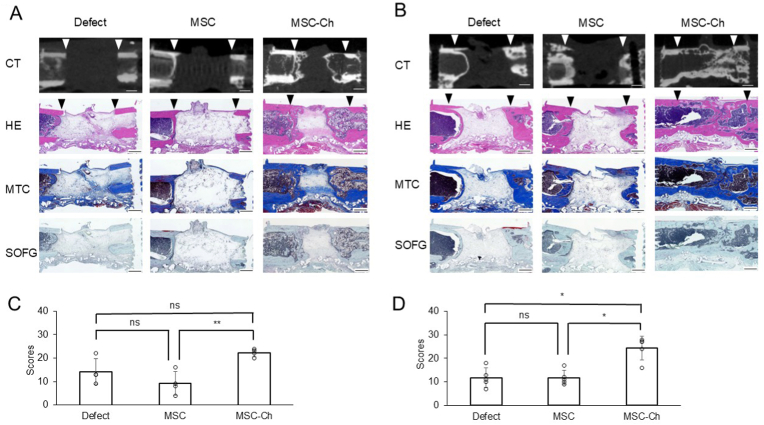
Histological findings with corresponding CT images and scoring at 6 and 12 weeks post-implantation (A) Histological findings and corresponding CT slices at 6 weeks post-implantation. (B) Histological findings and corresponding CT slices at 12 weeks post-implantation. (C) Histological scoring at 6 weeks post-implantation. (D) Histological scoring at 12 weeks post-implantation. Downward-pointing triangles denote the resected bone margins, with the intervening region representing the bone-defect area. CT, computed tomography; H&E, haematoxylin and eosin staining; MSC, mesenchymal stromal cell; MSC-Ch, mesenchymal stromal cell-derived chondrocyte; MTC, Masson's trichrome staining; ns, non-significant; SOFG, Safranin O and Fast Green staining. Scale bars = 1 mm (A, B). ∗*p* < 0.05, ∗∗*p* < 0.01

### 3.8 Histological analysis

Fig. 5A and B shows representative histological specimens obtained at 6 and 12 weeks post-implantation, respectively. At 6 weeks post-implantation, new bone formation in both the Defect and MSC groups was limited to the bone resection stumps. In contrast, the MSC-Ch group exhibited enhanced cortical and cancellous bone formation predominantly originating from the proximal femoral resection stump. Cartilage tissue was also observed in some areas of defect in the MSC-Ch group. By 12 weeks post-implantation, the defects in the Defect and MSC groups were largely filled with fatty and fibrous tissue. Conversely, the MSC-Ch group demonstrated that the defect was bridged by newly formed bone. Notably, the MSC-Ch group was largely free of features suggestive of chronic inflammation, such as inflammatory cell aggregates and fibrosis, at both time points. The histological findings at 24 weeks post-implantation are shown in Supplementary Fig. 2B.

Fig. 5C and D presents the results of histological scoring at 6 and 12 weeks post-implantation, respectively. At 6 weeks post-implantation, the histological scores were 11.7 ± 2.3 in the Defect group, 11.0 ± 4.4 in the MSC group, and 21.7 ± 1.5 in the MSC-Ch group (n = 4 per group; *p* = 0.007). By 12 weeks post-implantation, these respective scores were 11.8 ± 4.1 in the Defect group, 11.8 ± 3.1 in the MSC group, and 24.4 ± 4.9 in the MSC-Ch group *(*n = 5 per group; *p* < 0.001). This is consistent with the micro-CT results. Interestingly, the Defect and MSC groups showed scores indicating no cartilage formation (Supplementary Figs. 3 and 4).

**Fig. 6:**
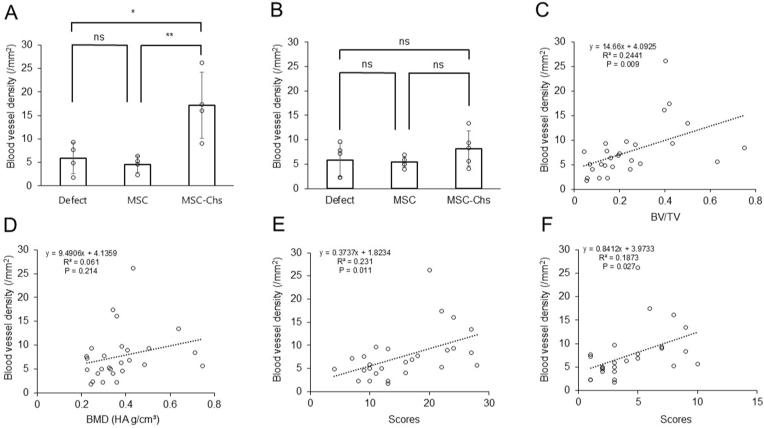
Vessel density in the bone defect area and its correlation with CT and histological scoring. (A) Vessel density at 6 weeks post-implantation. (B) Vessel density at 12 weeks post-implantation. (C) Correlation between vessel density and BV/TV. (D) Correlation between vessel density and BMD. (E) Correlation between vessel density and the total histological score. (F) Correlation between vessel density and the new bone (NB) histological score. BMD, bone mineral density; BV/TV, bone volume/total volume; MSC, Mesenchymal stromal cell; MSC-Ch, mesenchymal stromal cell-derived chondrocyte; NB, newly formed bone; ns, non-significant.

Fig. 6A and B shows the vessel density at 6 and 12 weeks post-implantation. At 6 weeks, the vessel density was statistically significantly higher in the MSC-Ch group (17.1 ± 7.1 vessels/mm^2^) compared to the Defect group (5.9 ± 3.3 vessels/mm^2^) and the MSC group (4.6 ± 1.7 vessels/mm^2^) (n = 4 per group; p = 0.007). At 12 weeks, the densities were 5.8 ± 3.4, 5.4 ± 1.1, and 8.2 ± 3.6 vessels/mm^2^ in the Defect, MSC, and MSC-Ch groups, respectively (n = 5 per group; p = 0.309). Correlation analysis revealed a positive correlation between vessel density and several parameters, including BV/TV, the total histological score, and the score for NB (Fig. 6C–E), whereas no significant correlation was observed with BMD (Fig. 6E). These results suggest a strong association between angiogenesis and bone regeneration.

### 3.9 Undecalcified stained sections

**Fig. 7:**
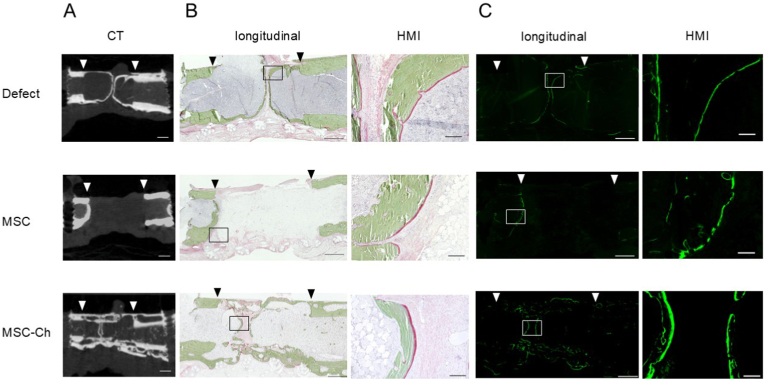
Histological analysis using Villanueva–Goldner staining and calcein fluorescence, shown with corresponding CT images. (A) CT images. (B) Villanueva–Goldner staining results. (C) Calcein fluorescence results. Downward-pointing triangles denote the resected bone margins, with the intervening region representing the bone defect area. CT, computed tomography; HMI, high-magnification image; MSC, Mesenchymal stromal cell; MSC-Ch, mesenchymal stromal cell-derived chondrocyte. Scale bars = 1 mm (longitudinal) and 100 μm (HMI).

Evaluation using non-decalcified specimens was performed on femurs harvested at 8 weeks post-implantation. This time point was selected as optimal for this specific analysis because bone formation might still be limited at 6 weeks, whereas at 12 weeks, the majority of the defect could potentially be replaced by mature bone tissue, with the peak period of active osteogenesis possibly having passed. In Villanueva–Goldner staining, osteoid-stained red was observed in the newly formed bone within the defect sites in both the Defect and MSC-Ch groups. However, in the MSC group, the osteoid was confined primarily to the periphery of the osteotomy margins (Fig. 7B). Furthermore, calcein fluorescence, which is indicative of active mineralisation fronts, was evident in the MSC-Ch group (Fig. 7C). The percentage of the calcein-positive area within the bone defect was 0.54 %, 0.37 %, and 2.76 % for the Defect, MSC, and MSC-Ch groups, respectively (n = 1 per group).

## 4 Discussion

This study demonstrates that scaffold-free cartilage constructs fabricated using a bio-3D printer significantly promote CSD healing. The key novelty of this research lies in demonstrating the efficacy of AT-MSCs within a bone defect repair model that recapitulates endochondral ossification. Although previous studies have reported that chondrogenically induced AT-MSCs can promote healing of mandibular bone defects [23], to the best of our knowledge, this is the first study to successfully regenerate a long bone CSD using AT-MSCs as the cell source by mimicking the endochondral ossification pathway. The cartilage constructs generated in this study exhibited characteristics similar to those of native cartilage, as confirmed by evaluations of the extracellular matrix (Safranin O and type II collagen immunostaining), morphology (electron microscopy), and mechanical properties. Consistently, both CT and histological analyses confirmed that the implantation of these cartilage constructs markedly accelerated the healing of femoral defects in rats. This finding suggests novel possibilities for the application of AT-MSCs in BTE.

### 4.1 Cell source

BM-MSCs and SM-MSCs exhibit higher chondrogenic potential than AT-MSCs [10]. Furthermore, these cells expressed higher levels of type X collagen [24]. Therefore, BM-MSCs and SM-MSCs have been more commonly used as cell sources in studies aimed at mimicking endochondral ossification. However, the use of BM-MSCs or SM-MSCs faces challenges such as the need for invasive harvesting procedures and low cell yields. This necessitates prolonged *in vitro* expansion to obtain sufficient cells for therapeutic applications [25]. Despite these reported disadvantages, AT-MSCs were selected as the cell source for the present study for four primary reasons. First, recent studies have significantly improved their chondrogenic differentiation potential. Approaches such as supplementation with growth factors, application of centrifugal force, and exposure to hypoxic conditions have all been shown to enhance chondrogenesis. Specifically, growth factors such as TGF-β and BMP potently induce SRY-box transcription factor 9 (SOX9), the master transcription factor for chondrogenesis [10]; centrifugal force has also been reported to upregulate *SOX9* expression [11]; and hypoxia promotes chondrogenic potential via the hypoxia-inducible factor 1-alpha (HIF-1α) pathway [12]. Second, we confirmed the capacity of AT-MSCs to undergo endochondral ossification after chondrogenic differentiation in our own preliminary experiments. In an *in vitro* model mimicking this process, the engineered cell constructs showed significant calcification, achieving a compressive strength of 12 MPa, comparable to cancellous bone (Supplementary Fig. 5). This finding demonstrates that AT-MSCs can not only differentiate into cartilage but also form bone-like tissue *in vitro*, supporting their suitability for our study and aligning with our *in vivo* results. However, the calcification pattern was heterogeneous *in vitro*, in contrast to the uniform calcification observed *in vivo*, likely due to the difficulty of perfectly replicating the complex *in vivo* environment. Third, this study was designed with a primary focus on clinical application. Our bio-3D printer requires a large number of cells to fabricate three-dimensional constructs. AT-MSCs can be harvested in large quantities through a relatively minimally invasive procedure, whereas clinically harvesting sufficient BM-MSCs or SM-MSCs is practical. Similarly, while induced pluripotent stem cells (iPSCs) have been explored as a useful source for cartilage regeneration, their clinical application is limited by complex preparation and prohibitive costs with mass production [26]. Fourth, we prioritised the use of an autologous implantation model. Bone regeneration involves various immune reactions, and it has been reported that normal bone remodeling is impaired in xenogeneic implantation models [27]. Therefore, it was crucial to conduct our investigation in a natural immune environment. Establishing an autologous model by harvesting sufficient BMSCs or SMSCs from rats is exceedingly difficult. This study strongly indicates that AT-MSCs are a promising cell source for BTE approaches designed to mimic endochondral ossification.

### 4.2 Chondrogenically induced versus non-induced constructs in promoting CSD healing

While some studies have reported that non-induced AT-MSCs can promote CSD healing, these investigations predominantly utilised models of anatomical sites characterised by a high proportion of cancellous bone and abundant blood supply, such as the calvaria or femoral condyles [6,28]. In contrast, the femoral diaphysis is a challenging segmental bone defect model where, consistent with previous reports, constructs without prior chondrogenic induction often fail to achieve union [29,30]. Our findings are consistent with this, as the MSC group, composed of undifferentiated AT-MSCs, resulted in non-union. This outcome is likely due to three factors: (1) poor cell survival in the poorly vascularized environment, (2) residual micromotion from the soft and deformable constructs, which is known to inhibit bone healing, and (3) insufficient osteogenic signalling, as the surrounding muscle and adipose tissues at the implantation site may not have provided uniform differentiation cues. This outcome underscores the significant advantages of pre-implantation chondrogenic induction for bone healing in such challenging environments. Specifically, chondrocytes exhibit greater tolerance to hypoxia than MSCs or osteoblasts and demonstrate significantly higher viability under avascular conditions, thereby circumventing problems associated with poor vascularisation [3]. Additionally, hypertrophic chondrocytes can promote angiogenesis and osteogenesis by releasing inductive factors such as vascular endothelial growth factor and BMPs [3]. Indeed, our study confirmed this, as the MSC-Ch group showed a significantly higher number of blood vessels at 6 weeks post-implantation, and a strong correlation was observed between vessel quantity and new bone formation. We propose that this successful healing proceeds via an endochondral ossification pathway. The process begins when the implanted cartilage construct integrates within the hematoma and acts as a scaffold. Chondrocytes subsequently release pro-angiogenic factors like VEGF, inducing vascular invasion from the host tissue—a step strongly supported by the increased vessel density we observed. Once vascularized, the rise in oxygen tension triggers chondrocyte hypertrophy and matrix calcification. This calcified cartilage serves as a template for new bone, which is subsequently resorbed by osteoclasts and replaced by osteoblasts depositing new bone matrix. Our histological results, showing cartilage being replaced by new bone, together with the strong positive correlation between vessel density and bone formation parameters (BV/TV and histological scores), are consistent with this proposed mechanism and with previous reports [31].

Clinically, CSD resulting from trauma or infection often involves damage to the surrounding soft tissues, including the periosteum and muscles, leading to a suboptimal environment for bone healing. Consequently, there is a critical need for therapeutic strategies that can effectively promote bone union even under demanding conditions. In this context, implantation of chondrogenically primed constructs represents a rational therapeutic approach.

### 4.3 Advantages of scaffold-free cartilage constructs for endochondral ossification *in vivo*

Scaffold-free techniques offer the advantage of circumventing problems associated with scaffold use, such as chronic inflammation, fibrosis, and biofilm formation during infection. Furthermore, some studies suggest they may achieve self-organisation more rapidly than scaffold-based approaches [13]. Conversely, the primary benefits of scaffold-based techniques are their precise geometric control and high initial stability post-implantation due to their mechanical properties [14]. For instance, beta-tricalcium phosphate (β-TCP), one of the most frequently employed materials in clinical practice, provides these advantages; its sufficient rigidity facilitates handling and ensures initial stability upon implantation [32]. However, a significant limitation of β-TCP is its lack of intrinsic osteoinductive properties, rendering it unsuitable for effective regeneration of large bone defects [32]. Indeed, in supplementary experiments conducted in the present study where β-TCP (NEOBONE; CoorsTek, Inc., Tokyo, Japan) was implanted into bone defects, cell infiltration into the scaffold material was limited, and complete bone healing was not achieved (Supplementary Fig. 6). Furthermore, other studies have shown that bone union is not achieved even when cells are combined with scaffold materials [29,30].

In contrast, novel scaffolds reported in recent years have demonstrated superior osteogenic potential. In studies focusing on cellular metabolic activity, it has been reported that the degradation products of scaffolds themselves function as intermediates or coenzymes in the tricarboxylic acid cycle, or promote bone regeneration by elevating mitochondrial membrane potential [33,34]. These findings offer insights into strategies for maintaining cellular activity within the harsh microenvironment of bone defects. While these studies employed a strategy of exogenously supplying metabolic intermediates as energy sources, we postulate that in our study, bone regeneration was promoted by the release of VEGF from the cartilage constructs implanted into the bone defects during endochondral ossification, which induced rapid vascular invasion by the host [31]. In essence, it can be inferred that the maintenance of cellular activity was enabled by the rapid recruitment of an energy supply from the host. In addition, a study reporting that bone regeneration was promoted by recapitulating condensation—a key process in bone development—demonstrated that cell aggregation enhances E-cadherin-mediated cell–cell interactions. This interaction induces an intracellular metabolic shift, thereby improving the stability and translational efficiency of the Runx2 protein [35]. Interestingly, both that study and our present research share the common methodological approach of cell aggregation. Furthermore, similar to osteogenic differentiation, it has been demonstrated that cell aggregation promotes chondrogenic differentiation by upregulating N-cadherin expression [36]. We suggest that in our approach utilising a bio-3D printer, N-cadherin expression was upregulated through cell aggregation and the fusion of cellular aggregates, potentially enhancing chondrogenic potential. These novel scaffolds possess the advantages of being functionalised with superior properties, as well as offering ‘off-the-shelf’ convenience regarding storage and transport. They can be deployed rapidly when required and are relatively easy to handle during surgical procedures. Conversely, although our method entails significant time and cost to culture large quantities of cells and fabricate cellular constructs, it eliminates the risks associated with scaffold use, such as inflammation, acidosis, and fibrosis. Moreover, the absence of a scaffold offers the distinct advantage of allowing for more natural and rapid self-organisation.

Supplementing scaffolds with BMPs to impart osteoinductive properties significantly enhanced osteogenesis. Combination products featuring scaffolds and BMPs have been clinically utilised in some countries, and their effectiveness in promoting bone union has been demonstrated. However, significant concerns regarding the potential complications (e.g., ectopic bone formation, bone resorption secondary to severe inflammatory responses, wound dehiscence, and infection) persist [37].

Representative scaffold-free techniques for tissue engineering include the use of cell sheets, moulding methods, and fabrication of cartilage fragments. Although cell sheets exhibit biological activity, they often possess insufficient mechanical strength. It has been shown that they cannot completely heal CSD when applied independently [38]. Moulding methods offer classic and straightforward approaches. Cartilage constructs fabricated using these techniques exhibit osteogenic potential [39]. However, this approach is limited by challenges in nutrient perfusion and fabrication constraints, which restrict the construct's size and shape. Another technique is fabricating iPSCs-derived cartilage masses. Although successfully implanted in mouse CSD models [27], precise control over the construct's final geometry remains a challenge. In contrast, bio-3D printing enables precise control over the spatial positioning of cells and offers significant flexibility in designing construct geometry. Indeed, in a previous study, cartilage constructs were successfully generated to mimic the precise size and shape of native tissues based on CT data [16]. Leveraging these capabilities, supplementary experiments in the present study involved creating a relatively large (10 mm) beak-shaped CSD, and implanting a cartilage construct fabricated via bio-3D printing to mimic the geometry of the bone defect (Supplementary Fig. 7). Although bone formation was partially promoted, complete osseous union was not achieved (Supplementary Fig. 7E). Two main factors may explain the failure to achieve complete osseous union. The first factor concerns the surgical procedure, specifically the insufficient internal fixation of the plate. Because only a single screw can be inserted into the proximal bone fragment, this fragment may have experienced rotational instability around the screw axis. Achieving more robust fixation in future procedures may help promote complete osseous union. Furthermore, while we hypothesized that implanting two constructs side by side would promote healing by allowing vascular invasion into the gap between them, the optimal number and design of the constructs require further investigation. The second factor is the size of the defect, which may have prolonged the healing process. Histological analysis revealed the presence of chondrocytes within the defect area (Supplementary Fig. 7F), consistent with an intermediate stage of the healing process. This finding indicates that osseous union may be achieved over a longer time course. However, to achieve accelerate union, further mechanistic studies are needed to clarify the underlying causes, alongside refinements of both construct fabrication process and surgical implantation techniques. The high degree of flexibility in designing the shape and size of fabricable constructs afforded by bio-3D printing presents significant advantages for the treatment of CSD, which often requires customised solutions to match diverse patient-specific geometries. Open tibial fractures, in particular, are known to frequently result in substantial bone loss [1]. Moreover, many of these fractures are classified as complex, multi-fragmentary types (e.g. AO classification C3), often presenting intricate fracture site morphologies [40]. Bio-3D printing technology directly addresses this challenge by enabling the fabrication of various forms of cellular constructs that are precisely tailored to the specific geometry of individual CSD. Furthermore, the fabrication of larger constructs is theoretically feasible by employing printing designs that specifically address the need for adequate medium perfusion throughout the interior of the construct. To date, most *in vivo* studies investigating endochondral ossification strategies for bone regeneration have been confined to small animal models such as mice, rats, and rabbits [3]. This prevalence likely reflects inherent challenges in ensuring sufficient oxygen and nutrient delivery within larger scaled-up engineered tissues. Consequently, future investigations employing large animal models are necessary to rigorously evaluate the translational potential of these developmental engineering approaches before their clinical application.

### 4.4 Limitations

This study has several limitations. Firstly, we did not include a 'scaffold + cells' group as a control. Although we have preliminary data from a group where only the βTCP scaffold was implanted in two rats (n = 2), a future study with a direct comparison between our MSC-Ch group and a sufficiently powered 'scaffold + cells' group is warranted. Such a comparison would be essential to more clearly elucidate the specific advantages of our proposed method.

Secondly, the histological evaluation was performed on a single plane only. A 3D evaluation was conducted using CT, and in the Discussion section, it was noted that the histological and CT findings were largely consistent. However, a comprehensive histological evaluation using serial sections would be more ideal.

Thirdly, a limitation of this study was the 2 week period of cast immobilization. External fixation should be minimised, as it can influence bone remodeling. However, in a preliminary experiment without external fixation, plate failure occurred. Therefore, with the specific implants used in this study, immobilization was necessary to protect the graft site. A 2 week period was considered sufficient for construct engraftment and is consistent with protocols used in previous animal studies from our laboratory [41].

Fourthly, the current timeframe between initial cell harvesting and construct implantation spans approximately four weeks. Further optimisation of growth factor concentrations, oxygen concentration during culture, culture medium perfusion, and kinetics of inductive stimuli during the *in vitro* culture phase could potentially reduce this preparation duration.

Fifthly, this study did not include a detailed mechanistic analysis, such as gene expression profiling or signalling pathway investigations, to elucidate the precise mechanisms underlying the observed promotion of bone healing. We hypothesize that the implanted cartilage constructs promoted bone regeneration by inducing an endochondral ossification process, which involves vascular invasion, chondrocyte hypertrophy, subsequent calcification, and replacement by new bone. However, future studies are required to validate this proposed mechanism and to clarify the detailed molecular pathways involved.

Sixth, the mechanical properties of the regenerated bone were not systematically evaluated. Although a three-point bending test was performed on the regenerated bone in a single case from the MSC-Chs group at 12 weeks post-implantation, this was only a preliminary assessment. The resulting fracture load was 93.7 N, which was lower than that of the contralateral healthy femur (218N). This result highlights the need for more comprehensive mechanical testing in future studies. Furthermore, functional assessments pertinent to bone healing outcomes, such as gait analysis (e.g., evaluating walking distance and speed) or quantifying muscle mass in the treated limb, were not conducted. Characterizing the degree of functional recovery achieved because of accelerated bone regeneration represents an important direction for future investigation.

Finally, a limitation of this study is the relatively small sample size used for certain analyses. Specifically, statistical comparisons were precluded for the assessment of the calcein fluorescence area because of the limited number of specimens allocated for these particular evaluations. Future research with a larger sample size is required.

## 5 Conclusion

This study demonstrated that bio-3D printed, scaffold-free cartilage constructs derived from rAT-MSCs effectively promote the healing of CSD. This study highlights the therapeutic potential of AT-MSCs for bone defect regeneration by mimicking endochondral ossification.

## Declaration of generative AI and AI-assisted technologies in the writing process

During the preparation of this work the authors used Gemini 2.5 Pro to improve the readability of the text. After using this tool/service, the authors reviewed and edited the content as needed and take full responsibility for the content of the publication.

## Funding

This research was supported by Management Expenses Grants for 10.13039/100018366National University Corporations and JSPS KAKENHI (grant numbers 22K09383 and 24K15702).

## Declaration of competing interest

K.N. is a co-founder and shareholder of Cyfuse Biomedical K.K. and serves as Chief Technical Officer for Arktus Therapeutics. K.N. is also an inventor on patents related to the bio-3D printing technology used or described in this research, specifically Japanese Patent No. 4517125 (titled "Method for Production of Three-Dimensional Structure of Cells") and Japanese Patent No. 5896104 (titled "Cell structure production device"). The remaining authors declare that they have no competing interests.
