# Supplementary material for: A scaffold-free cartilage construct fabricated using a bio 3D printer accelerates critical-size bone defect regeneration
Source: J Orthop Translat. 2026 Feb 28;57:101033. doi: 10.1016/j.jot.2025.101033 (PMC12966593; doi:10.1016/j.jot.2025.101033)
Supplement: Multimedia component 1 [file mmc1.docx]

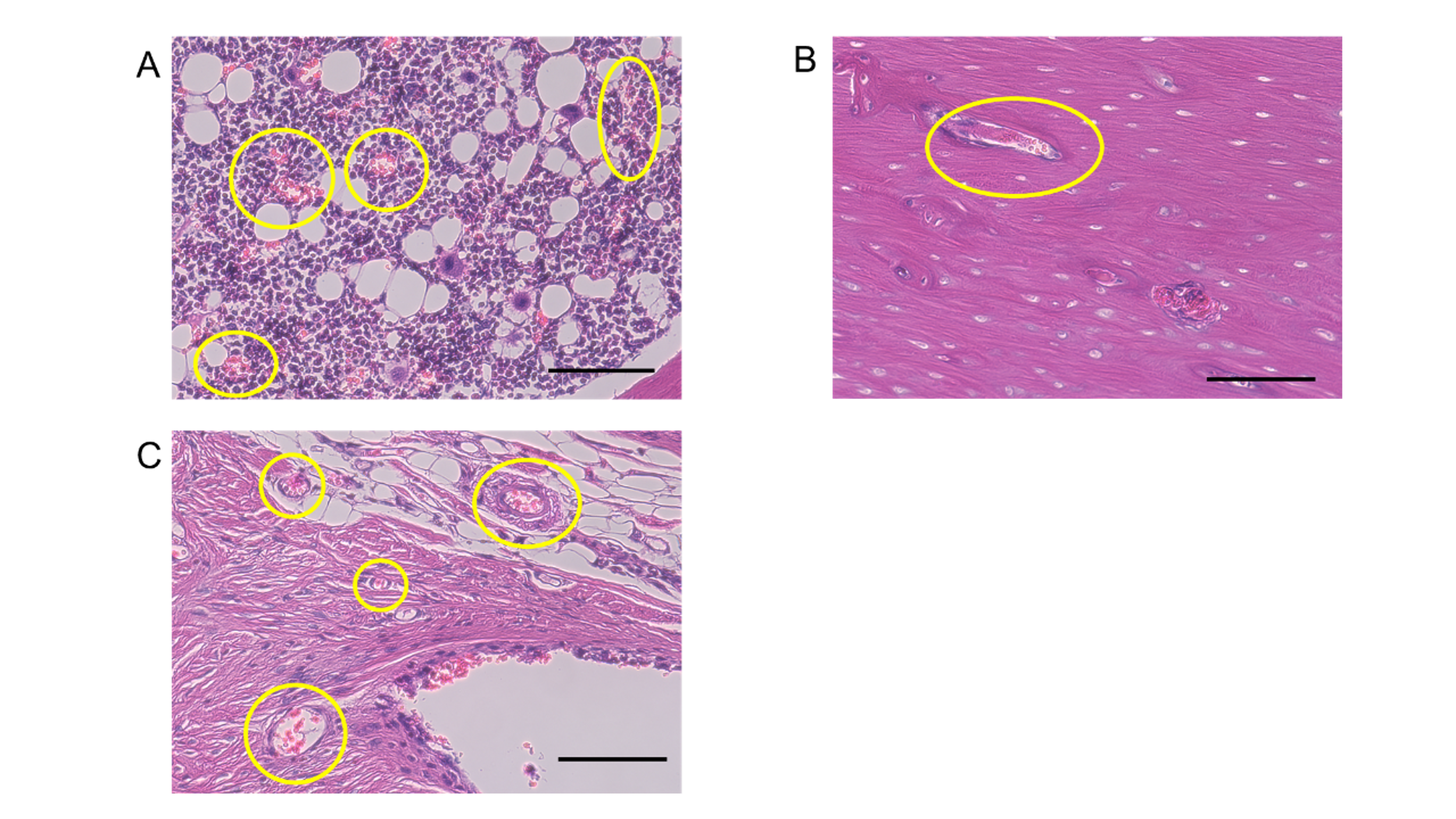


Supplementary Fig.1 Representative examples of vascular structures in rat femur for the measurement of vessel density.

(A) Blood vessels in cancellous bone.

(B) A blood vessel in cortical bone.

(C) Blood vessels in fibrous tissue.

Yellow circles indicate blood vessels. Note that not all vessels within the photographs are encircled.


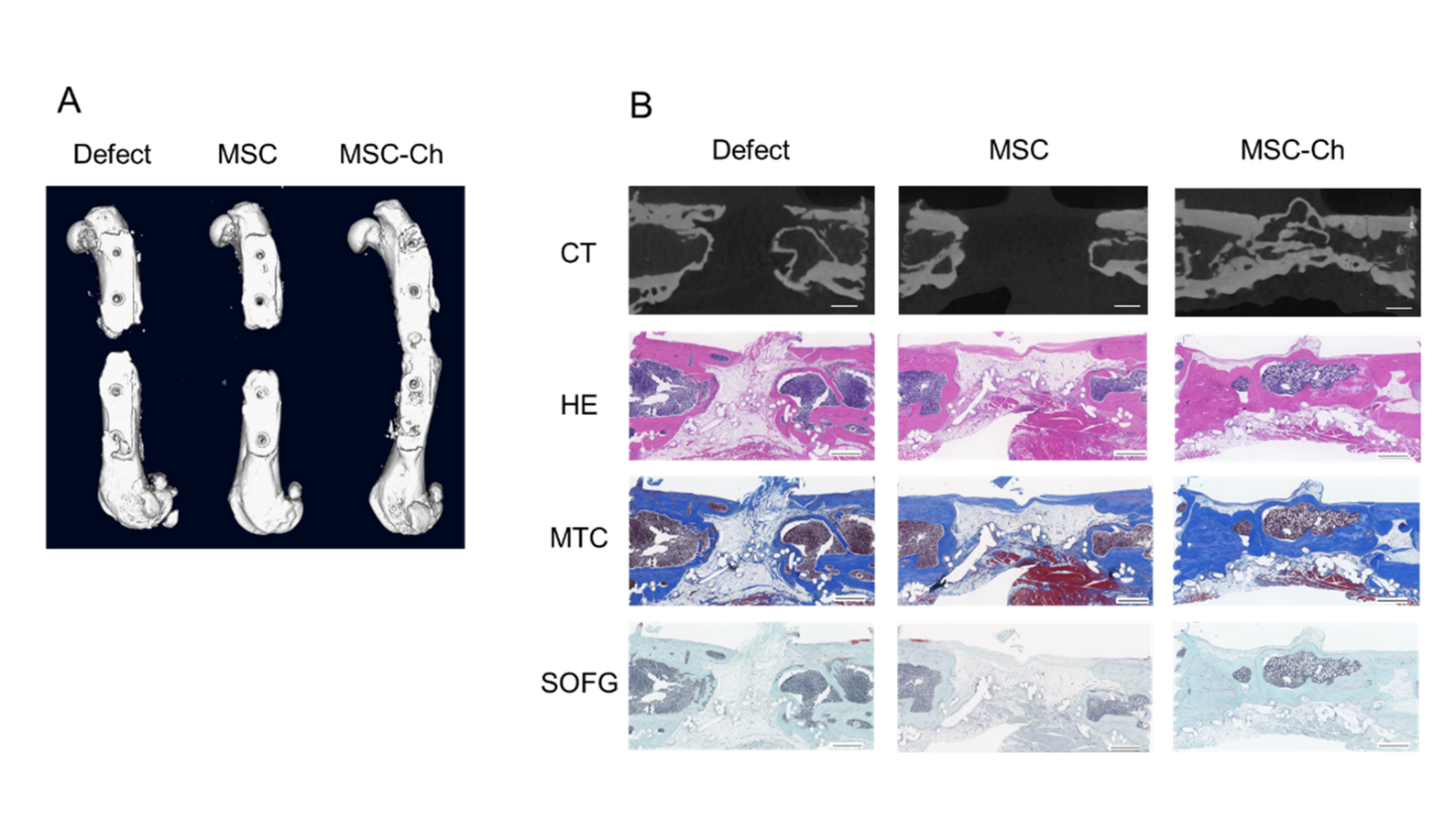


Supplementary Fig. 2. 3D-CT and histological evaluation at 24 weeks post-implantation.

(A) 3D-CT image at 24 weeks post-implantation.

(B) Histological findings at and corresponding CT slices 24 weeks post-implantation.

3D-CT, three-dimensional computed tomography; H&E, haematoxylin and eosin staining; MSC, mesenchymal stromal cell; MSC-Ch, mesenchymal stromal cell derived chondrocytes; MTC, Masson’s trichrome staining; SOFG, Safranin O and Fast Green staining


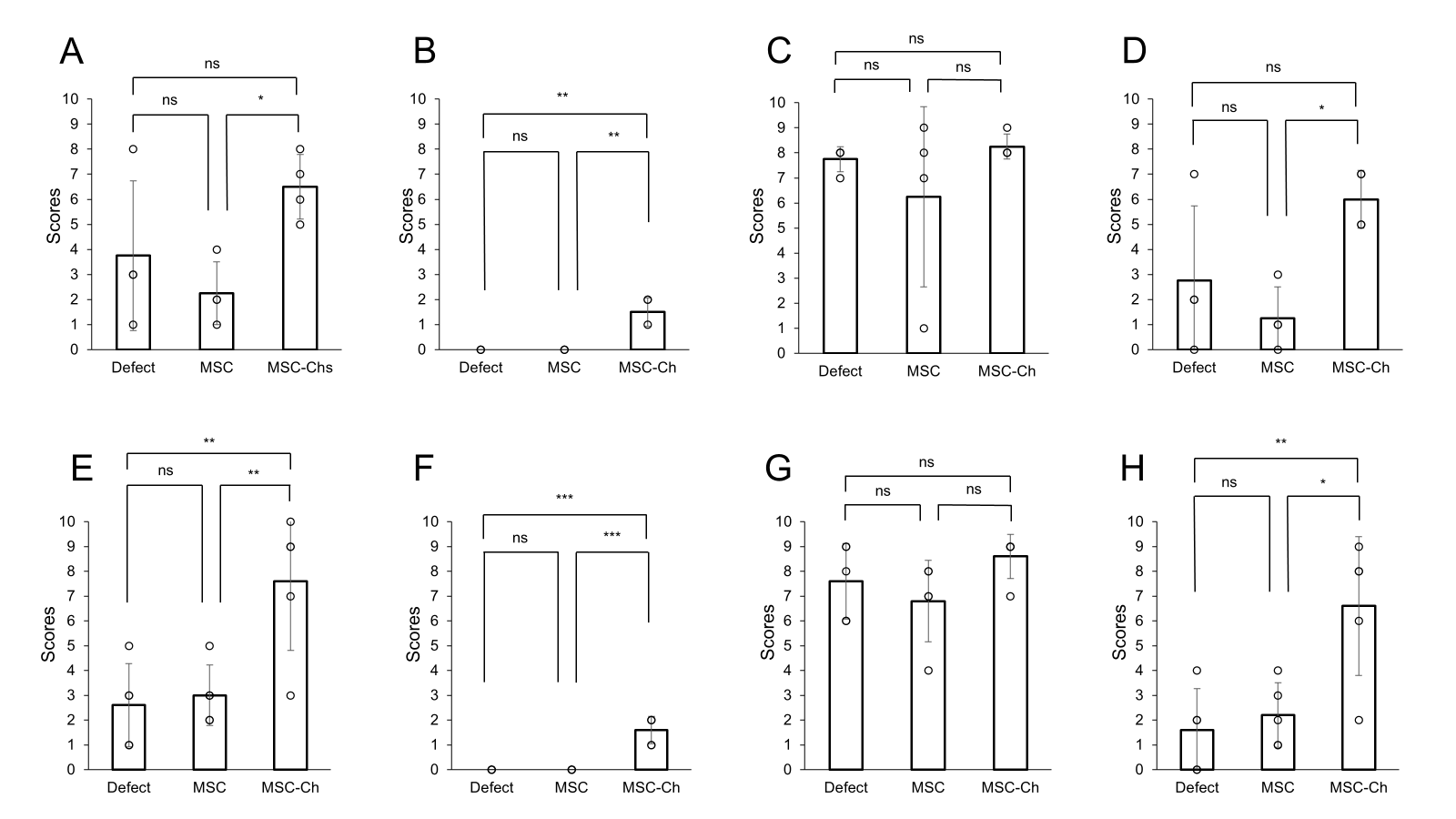


Supplementary Fig. 3. Results for each parameter of histological scoring.

(A) Newly formed bone at 6 weeks.

(B) Cartilage at 6 weeks.

(C) Fibrous tissue at 6 weeks.

(D) Remnant defect at 6 weeks.

(E) Newly formed bone at 12 weeks.

(F) Cartilage at 12 weeks.

(G) Fibrous tissue at 12 weeks.

(H) Remnant defect at 12 weeks.

* *p* < 0.05, ** *p* < 0.01, *** *p* < 0.001.

MSC, mesenchymal stromal cell; MSC-Ch, mesenchymal stromal cell derived chondrocytes; ns, non-significant.


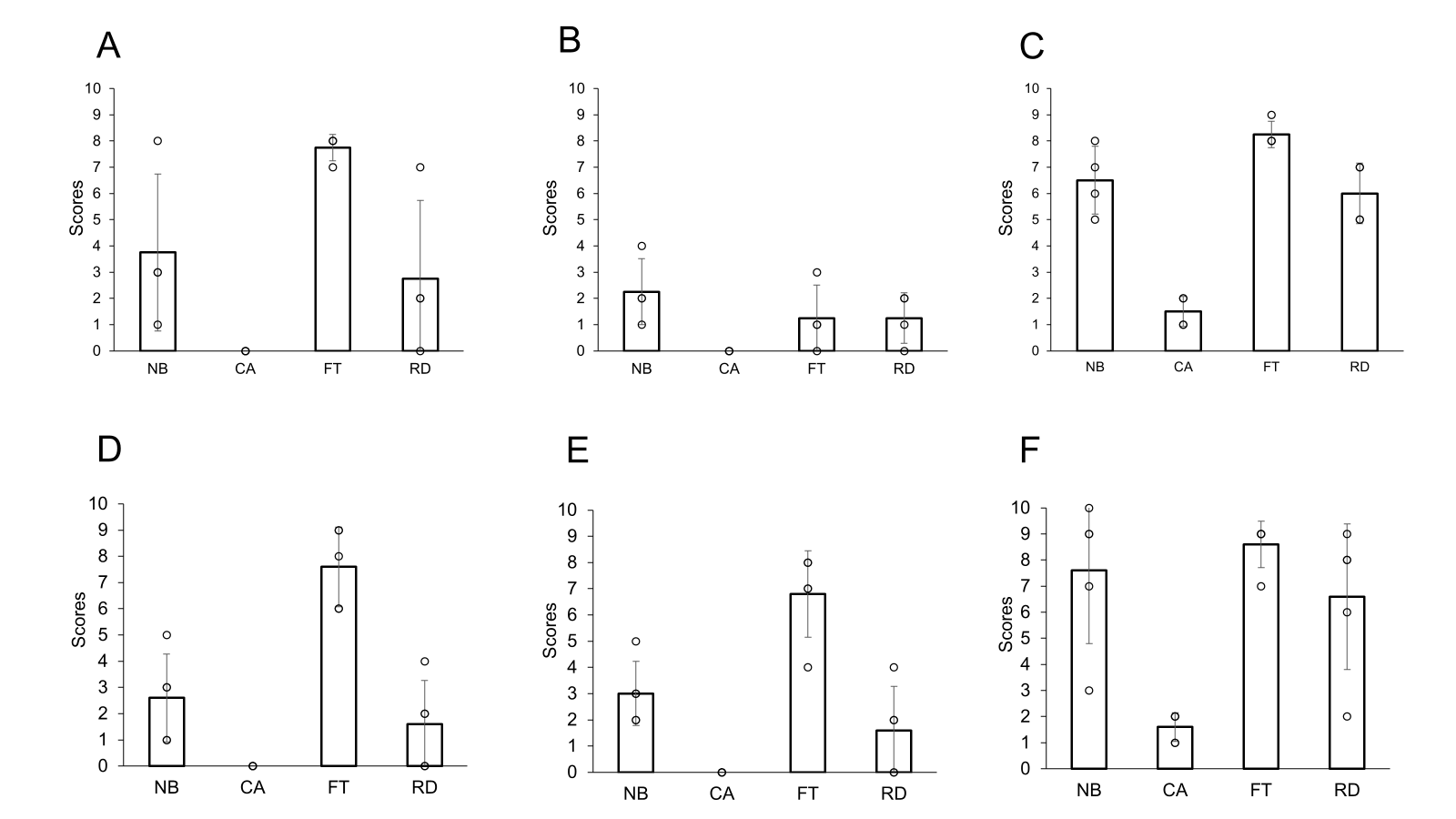


Supplementary Fig.4. Comparison of parameter-specific histological scores among the implantation groups.

(A) Defect group at 6 weeks post-implantation.

(B) ADSC group at 6 weeks post-implantation.

(C) ADSC-Ch group at 6 weeks post-implantation.

(D) Defect group at 12 weeks post-implantation.

(E) ADSC group at 12 weeks post-implantation.

(F) ADSC-Ch group at 12 weeks post-implantation.

CA, cartilage; FT, fibrous tissue; MSC, mesenchymal stromal cell; MSC-Ch, mesenchymal stromal cell derived chondrocytes; NB, newly formed bone; RD, remnant defect.


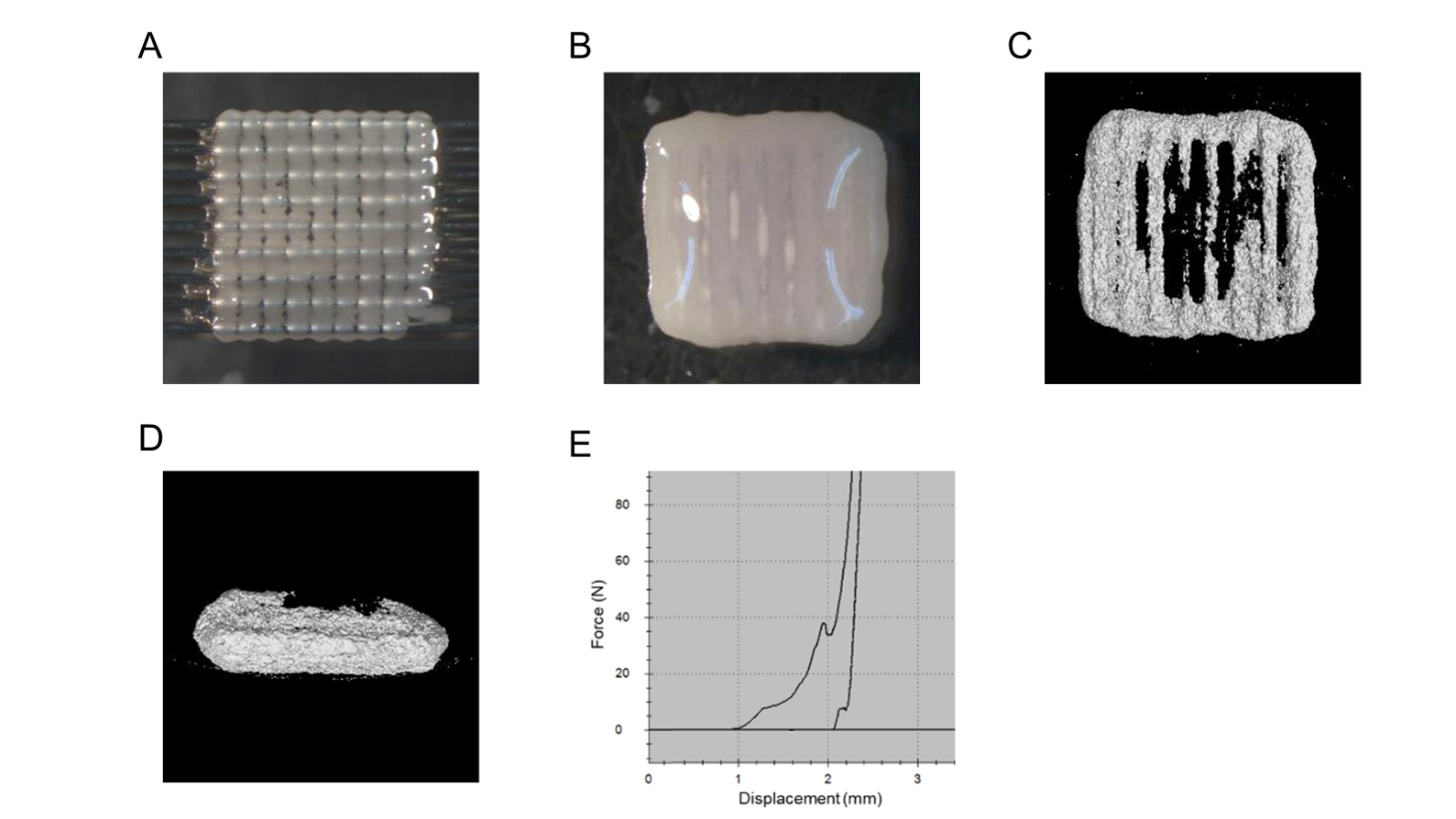


Supplementary Fig. 5 Bone-like tissue construct fabricated in vitro. The construct was cultured by switching to an osteogenic differentiation medium after the initial cartilage-like tissue construct was formed.

(A) Spheroids immediately after placement on the needle array.

(B) Gross appearance of the bone-like tissue construct.

(C) Frontal view of the construct by 3D-CT imaging.

(D) Lateral view of the construct by 3D-CT imaging.

(E) Result of compression test. The failure load of the construct was 37.9 N, corresponding to an ultimate compressive strength of 12.1 MPa.


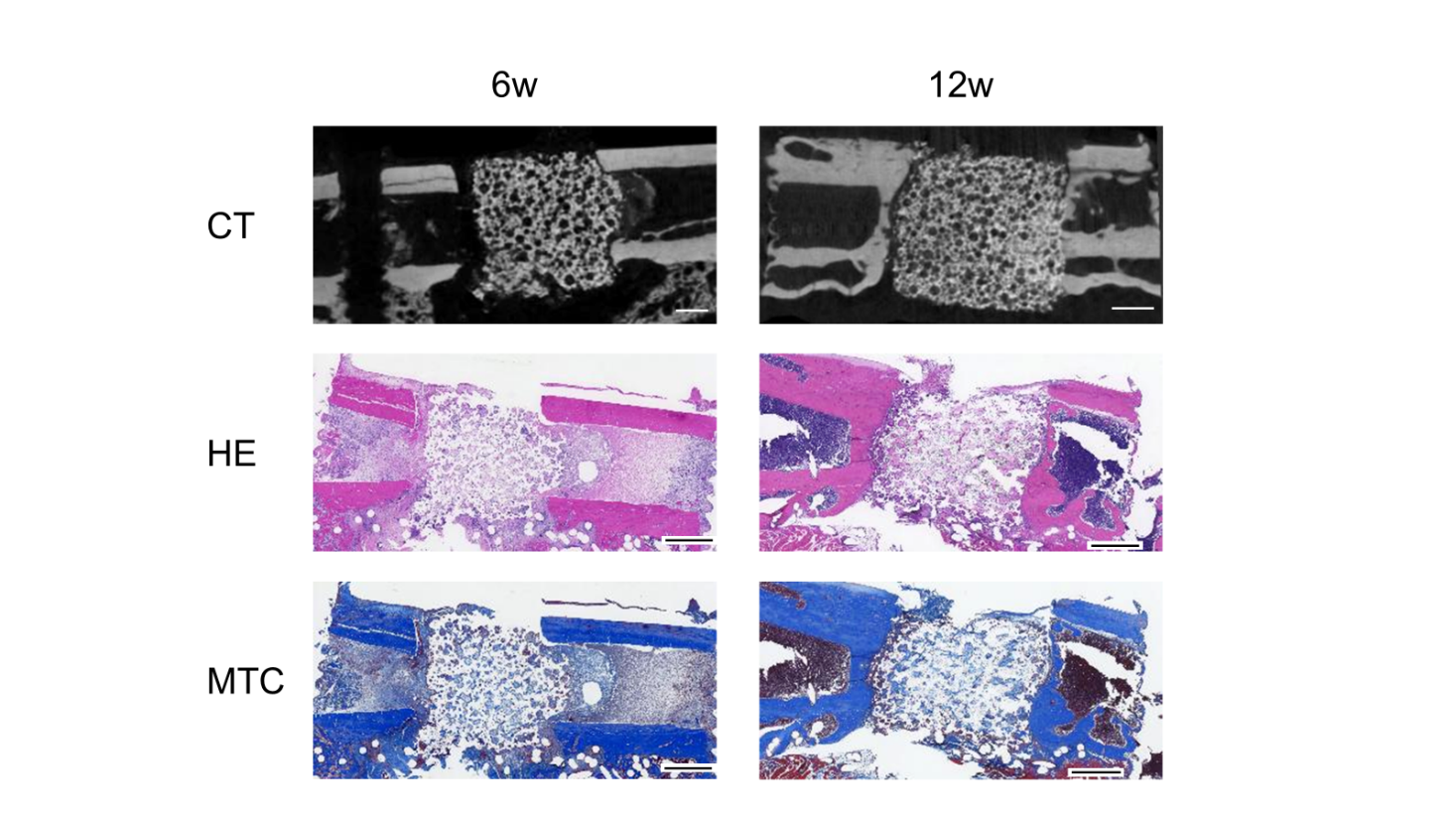


Supplementary Fig. 6. CT and histological images at 6 and 12 weeks post-implantation of a β-TCP block.

Scale bar = 1 mm.

β-TCP, beta-tricalcium phosphate; CT, computed tomography; H&E, haematoxylin and eosin staining; MTC, Masson’s trichrome staining


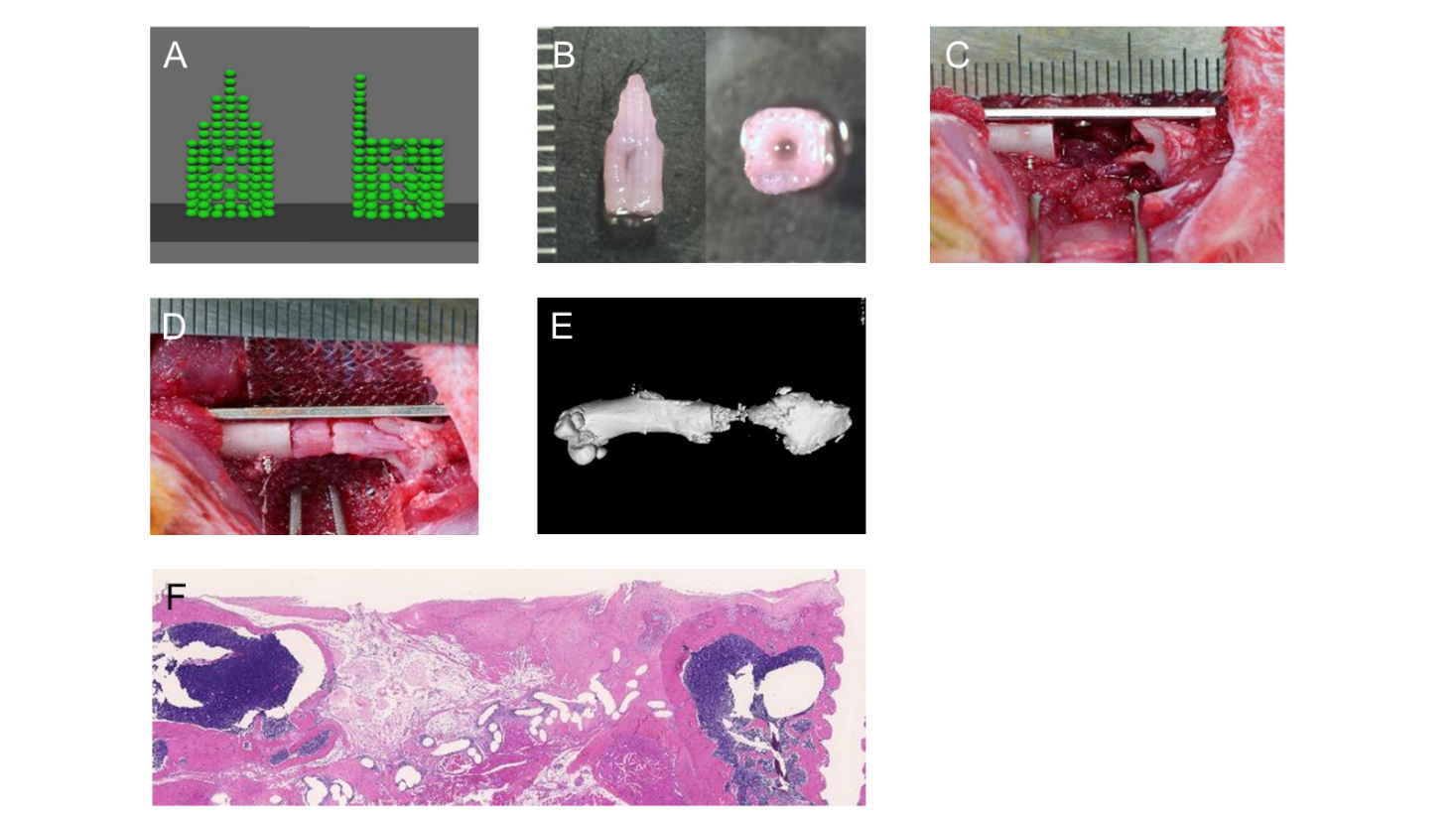


Supplementary Fig. 7. Fabrication and implantation of a cartilage construct tailored to the morphology of a beak-shaped bone defect.

(A) Computer design showing the arrangement of spheroids.

(B) Gross appearance of the cartilage construct.

(C) Beak-shaped bone defect.

(D) Two cartilage constructs implanted end-to-end in a 10-mm beak-shaped bone defect.

(E) 3D-CT images at 14 weeks post-implantation.

(F) Histological image at 14weeks post-implantation.

Each scale division corresponds to 1 mm (B, D). Scale bar = 1 mm(F).

3D-CT, three-dimensional computed tomography.
