# Supplementary material for: A scaffold-free cartilage construct fabricated using a bio 3D printer accelerates critical-size bone defect regeneration
Source: J Orthop Translat. 2026 Feb 28;57:101033. doi: 10.1016/j.jot.2025.101033 (PMC12966593; doi:10.1016/j.jot.2025.101033)

Supplemental Video 1.

Handling characteristics of a cartilage construct fabricated using a Bio-3D printer and cultured for 14 days.


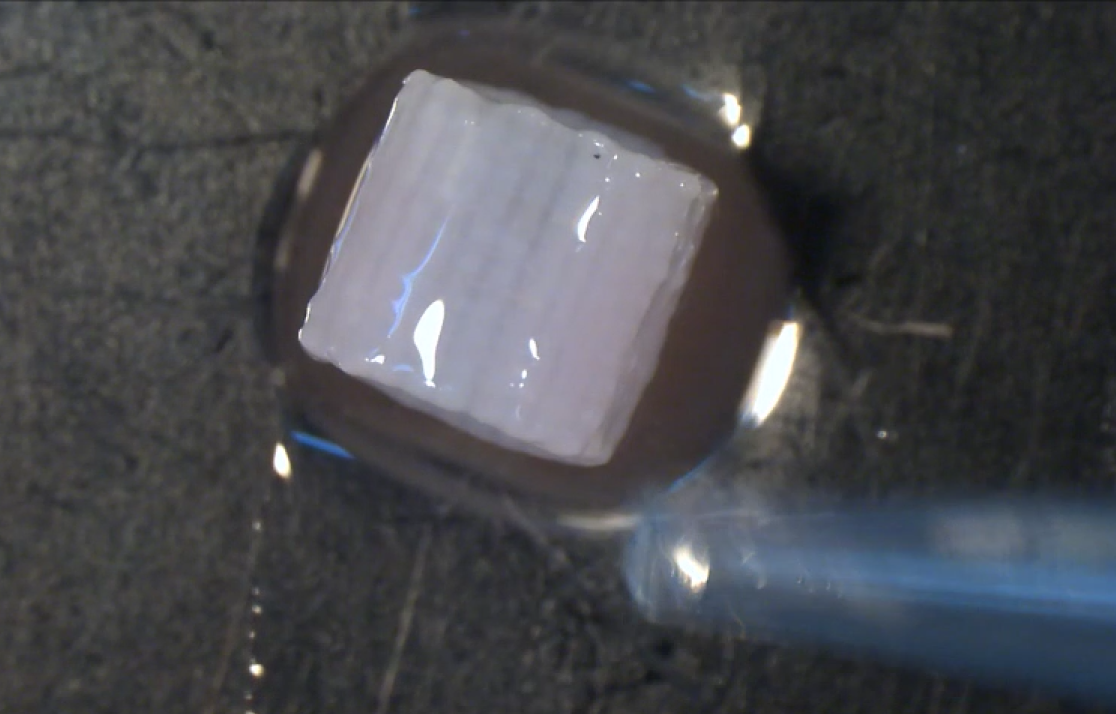

Supplement: Multimedia component 2 [file mmc2.docx]
